# Impaired Endothelial Function in Hereditary Angioedema During the Symptom-Free Period

**DOI:** 10.3389/fphys.2018.00523

**Published:** 2018-05-16

**Authors:** Davide Firinu, Pier P. Bassareo, Angela M. Zedda, Maria P. Barca, Antonio Crisafulli, Giuseppe Mercuro, Stefano Del Giacco

**Affiliations:** ^1^Unit of Internal Medicine, Department of Medical Sciences and Public Health, Allergy and Clinical Immunology, University of Cagliari, Cagliari, Italy; ^2^Unit of Cardiology and Angiology, Department of Medical Sciences and Public Health, University of Cagliari, Cagliari, Italy; ^3^Sports Physiology Lab., Department of Medical Sciences and Public Health, University of Cagliari, Cagliari, Italy

**Keywords:** hereditary angioedema, bradykinin, nitric oxide, asymmetric dimethylarginine, endothelium, atherosclerosis, flow mediated dilation

## Abstract

**Introduction:** The presence of coronary endothelial dysfunction was previously shown in Hereditary Angioedema (HAE) patients. The aim of our study was to evaluate the effect of HAE on systemic endothelial function and whether there was a relationship among endothelial function, asymmetric dimethylarginine (ADMA) -which is a strong inhibitor of nitric oxide synthesis-, and disease severity scores.

**Methods:** Twenty-four HAE patients (18 females, aged 47.9 ± 2 years) without factors known to interfere with endothelial function were studied and compared with 24 healthy peers age- and gender-matched. Endothelial function was assessed by means of non-invasive finger plethysmography (reactive hyperaemia index: RHI) and ADMA levels by high-performance liquid chromatography. HAE severity scores have been calculated according to published literature.

**Results:** In HAE patients RHI was lower (2.03 ± 0.46 vs. 2.82 ± 0.34, *p* < 0.0001) and ADMA higher (0.636 ± 7 vs. 585 ± 5 micromol/L, *p* < 0.01) than in controls. A statistically significant inverse correlation was revealed between RHI and patients' ADMA levels (*r* = −0.516, *p* = 0.009) as well as between RHI and patients' chronological age (*r* = −0.49, *p* = 0.015). A statistically significant correlation between RHI and ADMA was confirmed even when excluding the possible influence of cholesterol (*r* = −0.408, *p* = 0.048). No other significant correlations were found with the examined laboratory and clinical parameters (chronological age, age at disease onset, disease duration, severity scores, and gender).

**Conclusion:** The dysfunction previously shown in HAE patients at the coronary arteries seems to involve the peripheral vessels as well, without a correlation with disease severity.

## Introduction

Hereditary Angioedema (HAE) is a rare disease that is primarily caused by mutations in the *SERPING1* gene. This gene encodes for serine protease C1 inhibitor (C1-INH), with the HAE mutations resulting in quantitative (HAE type I, low C1-INH antigen levels) or functional deficiencies (HAE type II, normal C1-INH antigen levels); additionally, coagulation, fibrinolytic, complement and contact cascades are affected. This eventually leads to the overproduction of inflammatory molecules (Morgan, 2010; Longhurst and Cicardi, 2012; van Geffen et al., 2012), among which bradykinin (BK) plays a pivotal role (Nussberger et al., 1998; Cugno et al., 2003). BK is also involved in a subtype of HAE first recognized by Bork as HAE “type III” (Bork et al., 2000), and now named HAE with normal C1-INH function (Zuraw et al., 2012). A subgroup of patients bears mutations in the *F12* gene, and is defined as FXII-HAE (Firinu et al., 2015).

The main clinical HAE feature due to C1-INH deficiency and FXII-HAE is cutaneous or mucosal swelling, lasting between 1 and 5 days when untreated, and commonly involving the extremities, face, genitals, and gastrointestinal and respiratory tract (Zanichelli et al., 2015). Inflammatory BK may cause vasodilation and increased vascular leakage. The molecule binds to two distinct membrane receptors on endothelial cells: BK-receptor 1, inducible by proinflammatory cytokines, and BK-receptor 2, which is expressed constitutively and enhances vascular leakage (Kaplan et al., 2002).

In a study on coronary function in HAE patients, Demirtürk et al. showed the presence of early endothelial dysfunction, with development of atherosclerotic plaques (Demirtürk et al., 2012). A significant functional consequence of such endothelial damage is a reduction in the vasodilatory response to a range of pharmacological and physiological stimuli, such as reactive hyperemia. While endothelial function was previously assessed using only invasive techniques, non-invasive methods, such as the reactive hyperemia index (RHI), are currently available (Bassareo et al., 2010). Impaired endothelial function is correlated with future occurrence of adverse cardiovascular events and cardiac death (Celermajer et al., 1994).

Asymmetric dimethylarginine (ADMA) is a peptide in blood that is also a strong inhibitor of endothelial nitric oxide synthesis. High blood levels are associated with many pathological conditions related to atherosclerosis, including hypercholesterolemia, smoking, diabetes, hypertension, heart failure, chronic renal failure, erectile dysfunction, preeclampsia, and liver failure (Bassareo et al., 2014).

This study aimed to verify the presence of differences in RHI between HAE patients and healthy counterparts; to compare RHI in the two different subtypes of HAE included in the study; and to investigate the correlations between endothelial function in HAE patients and the laboratory and clinical parameters such as ADMA levels, lipid levels in blood, chronological age, age at disease onset, disease duration, severity scores, and sex.

## Materials and methods

### Study subjects

The study included 24 C1-INH-HAE or FXII-HAE patients (18 women, 6 men), with mean age at the time of the study 47.9 ± 2 years, mean age at disease onset 20.0 ± 1 years, and mean disease duration 27.8 ± 2 years, that were followed at the outpatients' clinic of Allergy and Clinical Immunology, University of Cagliari, Italy. Patients were examined during remission state, which were asymptomatic for at least 15 days before sampling.

Exclusion criteria were presence of pathological or environmental conditions, such as diabetes and smoking, that are known to interfere with endothelial functions and administration of drugs that could influence endothelial function, apart from those strongly needed for prophylactic therapy against life-threatening HAE attacks (Celermajer et al., 1992; Vapaatalo and Mervaala, 2001).

The results in the HAE group were compared with those in a control group comprising healthy peers, matched for age and sex.

This study was approved by the ethics committee of the University of Cagliari (*Number NP/2013/3226, protocol 692/2013*) and was conducted in accordance with the Declaration of Helsinki. All participants gave their informed written consent.

### Laboratory tests for HAE diagnosis

HAE was diagnosed by sequencing the *SERPING1* and *F12* genes (Firinu et al., 2013, 2015; Cicardi et al., 2014). Plasma levels of C1-INH antigen were measured using radial immunodiffusion (NOR Partigen C1-INH, Siemens Healthcare Diagnostics, Marburg, Germany); C4 antigen was measured using nephelometry. A chromogenic assay (Technochrom C1-Inhibitor, Technoclone, Vienna, Austria) was used to measure C1-INH activity. HAE severity scores were calculated according to published literature (Bygum et al., 2011; Gómez-Traseira et al., 2013).

### Endothelial function

Endothelial function was assessed using plethysmography-based probes placed on fingertips of the right hand (Endopath; Itamar Medical Ltd., Cesarea, Israel), a non-invasive, reliable, and reproducible method for quantifying RHI. The Endopath device allows measurement of changes in capillary diameter in the fingertips in response to an increase in shear stress (ischemia induced by occlusion of the brachial artery), which causes nitric oxide-dependent dilatation. The latter is reduced in a number of atherosclerosis-related pathological conditions (Bassareo et al., 2010). The strong reproducibility of this technique was demonstrated in a clinical study involving 19 centers in six European countries (Charakida et al., 2013). The clinical and predictive value of RHI measured at the fingertips is similar to that evaluated at the brachial artery (Zahedi et al., 2008).

This technique was approved by the American Food and Drug Administration as a diagnostic tool for use in the evaluation of endothelial function (Kuvin et al., 2003).

### Blood asymmetric dimethylarginine levels

One cm^3^ of blood was collected from the antecubital vein using a heparin injector. Blood concentration of ADMA was assessed using high-performance liquid chromatography with highly-sensitive laser fluorescent detection (Bassareo et al., 2012). This laboratory technique allows us to separate and quantify ADMA from deproteinized human plasma using a specific reagent. The same polymeric cation-exchange column was used for all samples (HAE patients and controls). This method proved to be highly sensitive, selective, and reproducible for determining ADMA levels, not only when using a commercial assay, but also when using a home-made kit (Valtonen et al., 2005).

#### Statistics

Non-parametric Mann Whitney *U*-test for non-continuous variables and chi-square test for continuous variables were performed. Univariate analysis, Pearson correlation coefficients, and regression lines for relationships between the various parameters were used as well. Multivariate analysis was not applied, because of the small sample size. However, partial correlation analysis was applied, in order to separate the possible influence of a variable on another one, when these two are deeply correlated, such as ADMA and age. The minimum level of statistical significance was set at *p* < 0.05 (software SPSS version 22.0, SPSS Inc., Chicago, Illinois, USA).

## Results

**Figure 1:**
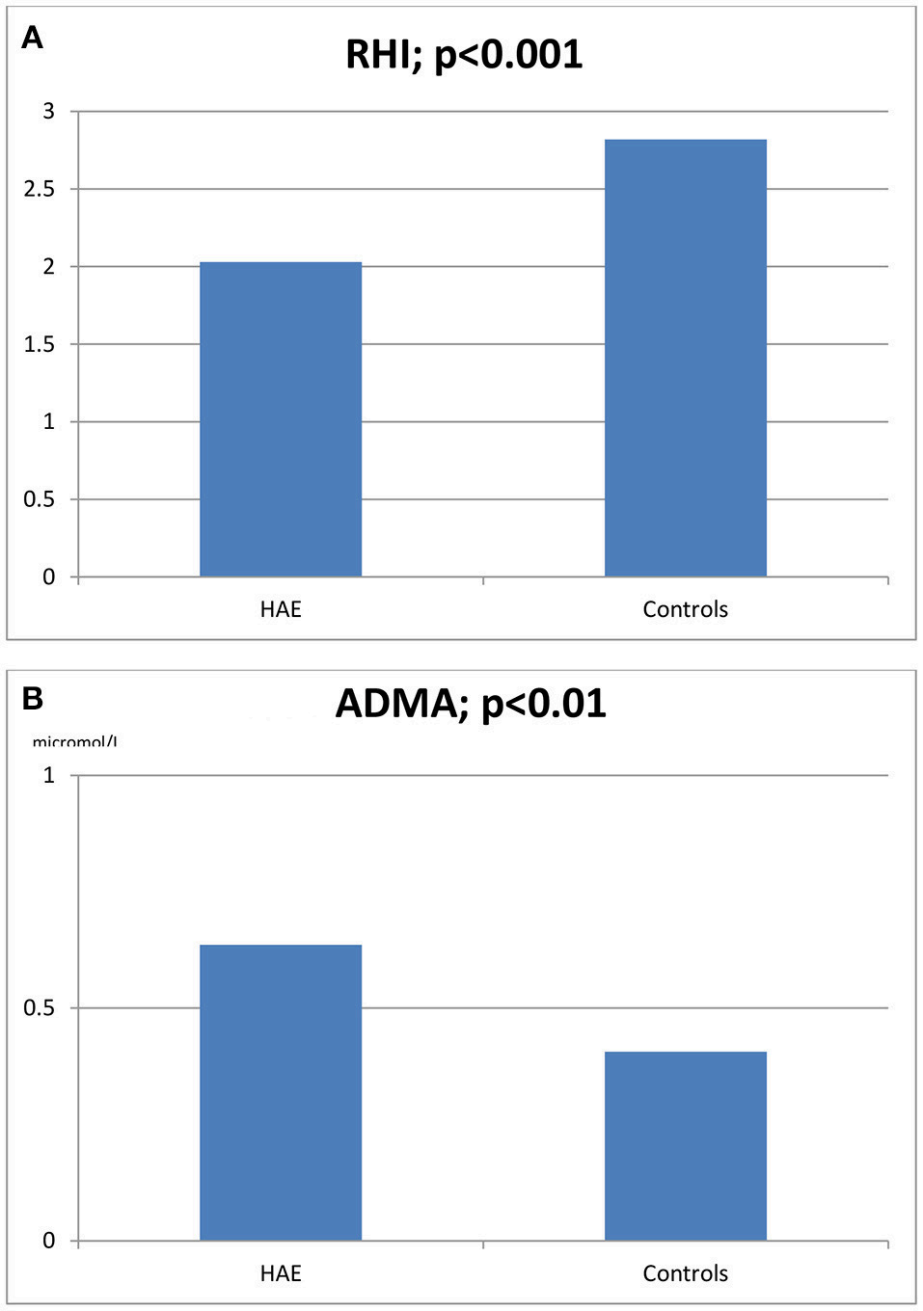
Reactive hyperemia index (RHI, **A**) and asymmetric dimethylarginine blood levels (ADMA, **B**) in HAE patients and healthy controls.

The characteristics of HAE patients and controls are summarized in Tables 1, 2. Statistically significant differences were detected for RHI (2.03 ± 0.46 vs. 2.82 ± 0.34, *p* < 0.0001) and ADMA (0.636 ± 7 vs. 585 ± 5 μmol/L, *p* < 0.01; see Figures 1A,B). When comparing RHI and ADMA in C1-INH-HAE and FXII-HAE subgroups, no statistically significant differences were found (2.02 ± 0.52 vs. 2.03 ± 0.38 and 0.640 ± 8 vs. 0.632 ± 6 μmol/L, both *p* = ns).

**Table 1:** Main clinical, laboratory data, and reactive hyperemia index results of patients affected by HAE studied with ENDOPAT.

**Patient id**	**RHI**	**AI (%)**	**HR**	Severity score^*^	**Gender**	**Age**	**Age at onset**	**Disease duration**	**C4**	**C1-INH Ag**	**C1-INH Fn%**
C1-INH-HAE 01	1,67	6	79	6	F	67	15	52	8	7,2	40
C1-INH-HAE 02	1,48	40	78	8	F	69	12	57	1	4,8	20
C1-INH-HAE 03	2,52	34	63	8	M	47	10	37	3	8	9
C1-INH-HAE 04	3,04	7	69	7	F	41	15	26	1	4,8	N.D.
C1-INH-HAE 05	2,47	2	68	7	F	54	10	44	3	7	33
C1-INH-HAE 06	2,41	−8	61	6	M	30	14	16	2	6	N.D.
C1-INH-HAE 07	2,56	5	64	4	F	31	19	12	N.D.	N.D.	N.D.
C1-INH-HAE 08	2,29	−9	82	4	F	29	18	11	N.D.	N.D.	N.D.
C1-INH-HAE 09	1,35	−4	71	0	M	40	N.A.	N.A.	5	6,5	N.D.
C1-INH-HAE 10	1,8	−16	97	0	M	44	N.A.	N.A.	6	8	N.D.
C1-INH-HAE 11	1,55	33	84	4	F	82	30	52	7	5,6	N.D.
C1-INH-HAE 12	1,74	32	66	6	M	47	11	36	5	6,4	15
C1-INH-HAE 13	1,49	18	86	7	F	52	12	40	2	5,6	22
C1-INH-HAE 14	1,97	33	70	6	F	48	15	33	1	7	27
**Patient id**	**RHI**	**AI (%)**	**HR**	**HAE-FXII score**^§^	**Gender**	**Age**	**Age at onset**	**Disease duration**	**C4**	**C1-INH Ag**	**C1-INH Fn%**
FXII-HAE 01	1,93	15	71	Asymptom	M	62	N.A.	N.A.	N.D.	N.D.	N.D.
FXII-HAE 02	1,6	−5	67	Severe	F	32	19	13	20	26,2	75
FXII-HAE 03	1,99	5	63	Moderate	F	38	14	24	19	22,2	N.D.
FXII-HAE 04	2,6	6	56	Moderate	F	31	23	8	14	17,4	85
FXII-HAE 05	1,93	−11	82	Mild	F	10	9	1	13	24	80
FXII-HAE 06	1,84	8	93	Severe	F	54	20	34	N.D.	N.D.	N.D.
FXII-HAE 07	2,1	9	60	Severe	F	43	33	10	13	28,1	80
FXII-HAE 08	2,01	29	64	Severe	F	58	21	37	16	26,8	N.D.
FXII-HAE 09	1,57	45	72	Mild	F	76	76	0	N.D.	N.D.	N.D.
FXII-HAE 10	2,77	70	69	Mild	F	66	24	42	N.D.	N.D.	N.D.

*RHI, reactive hyperemia index; AI%, augmentation index; HR, heart rate*.

* C1-INH-HAE score and

§ *HAE-FXII severity score calculated according to reference 17 and 18.Normal ranges as follows:C1-INH Antigen (Ag) 21–39 mg/dl; C1-INH Function (Fn) 70–130%; Serum C4 antigen 10–40 mg/dl. N.D., Not determined for this study; N.A., Not applicable*.

**Table 2:** Characteristics of patients, cardiovascular risk factors and specific HAE treatments of subjects enrolled in the study.

**Patient id**	**HAE type**	**Attenuated androgens intake**	**HAE prophylaxis**	**HAE on demand treatment**	**Current or previous treatment for dyslipidemia**	**Hypertension, heart failure, diabetes, metabolic syndrome, kidney disease**
C1-INH-HAE 01	C1-INH type I	No	None	Plasma derived C1-INH	No	No
C1-INH-HAE 02	C1-INH type I	No	Plasma derived C1-INH	Plasma derived C1-INH or icatibant	No	No
C1-INH-HAE 03	C1-INH type I	No	None	Plasma derived C1-INH	No	No
C1-INH-HAE 04	C1-INH type I	No	None	Plasma derived C1-INH or icatibant	No	No
C1-INH-HAE 05	C1-INH type I	No	None	Plasma derived C1-INH	No	No
C1-INH-HAE 06	C1-INH type I	No	None	Plasma derived C1-INH	No	No
C1-INH-HAE 07	C1-INH type I	No	None	Plasma derived C1-INH	No	No
C1-INH-HAE 08	C1-INH type I	No	None	Plasma derived C1-INH	No	No
C1-INH-HAE 09	C1-INH type I	No	None	Plasma derived C1-INH	No	No
C1-INH-HAE 10	C1-INH type I	No	None	Plasma derived C1-INH	No	No
C1-INH-HAE 11	C1-INH type I	No	None	Plasma derived C1-INH	No	No
C1-INH-HAE 12	C1-INH type I	No	None	Plasma derived C1-INH or icatibant	No	No
C1-INH-HAE 13	C1-INH type I	No	Plasma derived C1-INH	Plasma derived C1-INH	No	No
C1-INH-HAE 14	C1-INH type I	No	Plasma derived C1-INH	Plasma derived C1-INH or icatibant	No	No
FXII-HAE 01	FXII-HAE	No	None	Plasma derived C1-INH	No	No
FXII-HAE 02	FXII-HAE	No	Plasma derived C1-INH	Plasma derived C1-INH or icatibant	No	No
FXII-HAE 03	FXII-HAE	No	Plasma derived C1-INH	Plasma derived C1-INH or icatibant	No	No
FXII-HAE 04	FXII-HAE	No	None	Icatibant	No	No
FXII-HAE 05	FXII-HAE	No	None	Plasma derived C1-INH	No	No
FXII-HAE 06	FXII-HAE	No	None	Plasma derived C1-INH	No	No
FXII-HAE 07	FXII-HAE	No	None	Icatibant	No	No
FXII-HAE 08	FXII-HAE	No	None	Plasma derived C1-INH	No	No
FXII-HAE 09	FXII-HAE	No	None	Plasma derived C1-INH	No	No
FXII-HAE 10	FXII-HAE	No	None	Plasma derived C1-INH	No	No

A statistically significant correlation was found between RHI and ADMA (*r* = −0.516, *p* = 0.009), as well as between RHI and chronological age (*r* = −0.49, *p* = 0.015). A statistically significant correlation was confirmed even when excluding the possible influence of cholesterol level on the relationship between RHI and ADMA (*r* = −0.408, *p* = 0.048). No significant correlations were detected between RHI and sex, severity scores, age at disease onset, and disease duration (all *p* = ns).

## Discussion

Our findings revealed a significant decrease in endothelial function in HAE patients during the symptom-free period, when compared to a group of healthy peers. Furthermore, a strong correlation between RHI and ADMA was observed. While the pathological role in cardiovascular disease is somewhat unclear, ADMA is known to induce endothelial dysfunction, the earliest stage of atherosclerosis (Baum et al., 2016; Mangiacapra et al., 2016).

Our study was not designed to unravel the mechanisms behind the decrease in RHI and increase in ADMA, or their relationship. However, regarding a possible pathophysiological explanation, it might be hypothesized that both C1-INH-HAE and FXII-HAE subgroups had a shared endothelial dysfunction that was probably not caused by C1-INH deficiency or mutated coagulation FXII *per se*, but instead by bradykinin receptor-ADMA pathway activation. Although this metabolic pathway has not been adequately studied, ADMA levels increased after incubation with BK in a cellular model of human alveolar adenocarcinoma, while co-incubation with bradykinin B1 receptor inhibitor did not lead to a decrease in ADMA. This suggests that BK-dependent ADMA production may occur through bradykinin B2 receptor stimulation (Gamboa et al., 2015). Furthermore, previous studies showed that BK increases reactive oxygen species production through stimulation of NADPH oxidases; this in turn increases ADMA levels by increasing protein methylation while inhibiting ADMA degradation (Larsen et al., 2009; Luo et al., 2010). Another possible explanation for increased ADMA levels is the fact that BK may decrease dimethylarginine dimethylaminohydrolase activity, which is responsible for ADMA degradation (Gamboa et al., 2015). Again, C1-INH-HAE is able to dysregulate the activities of complement, coagulation, and contact systems (Kaplan and Joseph, 2014), and increased procoagulant and fibrinolytic activities were observed in HAE patients during attacks and remission phases (van Geffen et al., 2012; Reshef et al., 2015). The apparent thrombotic risk in patients with C1-INH-HAE, although not confirmed with clinical observations (Reshef et al., 2015), needs to be discussed in further studies, because of the endothelial dysfunction demonstrated in this work.

As none of the studied patients was taking attenuated androgens, our findings were not influenced by these drugs, which are known to impair lipid levels, thus leading to accelerated atherosclerosis (Széplaki et al., 2005). On the other hand, it was previously reported that in HAE subjects, most of the endothelial functions are normal in the inter-attack periods, as shown by normal blood levels of some markers of endothelial cell permeability (endothelin-1, von Willebrand factor) (Czúcz et al., 2012). However, increased endothelial nitric oxide synthase levels in attack-free periods were detected in C1-INH-HAE patients as well (Demirtürk et al., 2014; Costa et al., 2016).

More rapid development of coronary atherosclerosis in HAE patients was previously shown by altered coronary flow reserve measurement in the left anterior coronary artery. The latter is a non-invasive method useful for assessing coronary function, with results closely corresponding to invasive measurements (Caiati et al., 1999; Lethen et al., 2003). In a cohort of patients affected by C1-INH-HAE (most under long term prophylaxis with danazol), the coronary flow reserve was found to be decreased, even when the intima-media thickness in the carotid arteries was normal (Demirtürk et al., 2012). According to our findings, the early atherosclerosis detected with RHI was not related to disease severity scores or the duration of therapy (Demirtürk et al., 2012). Reduced coronary flow reserve is a sign of increased atherosclerosis, while reduced RHI is an early sign of atherosclerosis in peripheral vessels. The latter seems to occur more rapidly in HAE patients, in comparison with their healthy peers.

The present study has some limitations such as the small sample size. However, HAE is a rare disease, and groups of maximum 30 subjects were typically recruited in previous studies with similar design (Demirtürk et al., 2012; van Geffen et al., 2012; Wu et al., 2017). In addition, having studied HAE subjects only during inter-attack periods may have led to incomplete assessment of endothelial characteristics. Moreover, other factors potentially influencing endothelial response to ischemic stimuli, such as those that are usually administered for HAE attacks (plasma-derived C1-INH or icatibant), should be considered (Birjmohun et al., 2008). However, from an ethical point of view, it was obviously impossible to discontinue life-saving drugs in our patients.

In conclusion, this was the first study to report that the atherosclerotic process previously observed in coronary arteries also involves the peripheral vessels in HAE patients. Nitric oxide production impairment, through the still poorly-understood bradykinin receptor-ADMA pathway activation, was hypothesized to be involved (Rastaldo et al., 2007; Kim and Massett, 2016; Wang et al., 2016). This may indicate a much more extensive hardening of the arteries, involving the entire arterial tree. In practice, even though the main cause of death in HAE patients has been laryngeal involvement with subsequent asphyxia (25–30% of the patients in the first decades of life when untreated), the efficacy of the administered drugs has resulted in a decrease in mortality (0.35–0.5% in medically treated patients) (Varga and Farkas, 2008). In this respect, since atherosclerosis is a complex process that involves several mechanisms and is the leading cause of heart attacks, stroke, and peripheral vascular disease, regular cardiovascular follow-up is required in HAE patients (Penna et al., 2006; Yang et al., 2017).

## Author contributions

DF and PB: interpretation of the data and manuscript writing. AZ and MB: aquisition of the data. AC, GM and SD: final approval of the manuscript to be published.

### Conflict of interest statement

The authors declare that the research was conducted in the absence of any commercial or financial relationships that could be construed as a potential conflict of interest.
